# Histopathological grading of pediatric ependymoma: reproducibility and clinical relevance in European trial cohorts

**DOI:** 10.1186/1477-5751-10-7

**Published:** 2011-05-31

**Authors:** David W Ellison, Mehmet Kocak, Dominique Figarella-Branger, Giangaspero Felice, Godfraind Catherine, Torsten Pietsch, Didier Frappaz, Maura Massimino, Jacques Grill, James M Boyett, Richard G Grundy

**Affiliations:** 1 Dept. of Pathology, St. Jude Children's Research Hospital, Memphis, USA; 2 Dept. of Biostatistics, St. Jude Children's Research Hospital, Memphis, USA; 3 Dept. of Pathology, La Timone's Hospital, Marseille, France; 4 Dept. of Experimental Medicine and Pathology, University of Rome, Rome, Italy; 5 Laboratory of Pathology, St Luc Hospital, Bruxelles, Belgium; 6 Neuropathology Institute, University Clinic, Bonn, Germany; 7 Dept. of Oncology/Hematology, Centre Léon Berard, Lyon, France; 8 Division of Pediatrics, National Tumor Institute, Milan, Italy; 9 Dept. of Pediatric and Adolescent Oncology, Gustave Roussy Cancer Institute, Paris; 10 Children's Brain Tumor Center, University of Nottingham, Nottingham, UK

## Abstract

**Background:**

Histopathological grading of ependymoma has been controversial with respect to its reproducibility and clinical significance. In a 3-phase study, we reviewed the pathology of 229 intracranial ependymomas from European trial cohorts of infants (2 trials - SFOP/CNS9204) and older children (2 trials - AIEOP/CNS9904) to assess both diagnostic concordance among five neuropathologists and the prognostic utility of histopathological variables, particularly tumor grading.

**Results:**

In phase 1, using WHO criteria and without first discussing any issue related to grading ependymomas, pathologists assessed and independently graded ependymomas from 3 of 4 trial cohorts. Diagnosis of grade II ependymoma was less frequent than grade III, a difference that increased when one cohort (CNS9204) was reassessed in phase 2, during which the pathologists discussed ependymoma grading, jointly reviewed all CNS9204 tumors, and defined a novel grading system based on the WHO classification. In phase 3, repeat independent review of two cohorts (SFOP/CNS9904) using the novel system was associated with a substantial increase in concordance on grading. Extent of tumor resection was significantly associated with progression-free survival (PFS) in SFOP and AIEOP, but not in CNS9204 and CNS9904. Strength of consensus on grade was significantly associated with PFS in only one trial cohort (AIEOP). Consensus on the scoring of individual histopathological features (necrosis, angiogenesis, cell density, and mitotic activity) correlated with PFS in AIEOP, but in no other trial.

**Conclusions:**

We conclude that concordance on grading ependymomas can be improved by using a more prescribed scheme based on the WHO classification. Unfortunately, this appears to have utility in limited clinical settings.

## Background

Ependymoma is the third most common neuroepithelial tumor of the central nervous system (CNS) in childhood, after astrocytoma and medulloblastoma [1,2]. It currently presents a considerable therapeutic challenge, being incurable in more than half of cases. In contrast to the mainly spinal tumors of adults, childhood disease is dominated by intracranial tumors [1]. Treatment of pediatric intracranial ependymomas principally involves surgery and adjuvant radiotherapy, extent of surgical resection being a critical determinant of outcome [3]. The role of chemotherapy is controversial, but its use alongside radiotherapy has been the focus of several clinical trials, especially in the setting of attempts to avoid or to defer radiotherapy in infants [4-6,3].

The World Health Organization (WHO) classification of CNS tumors defines several histopathological variants of ependymoma [1]. Aside from the subependymoma (WHO grade I), which generally presents in adults and causes minimal morbidity, and very rare examples of intracranial myxopapillary ependymoma (WHO grade I), intracranial pediatric ependymomas are divided between classic (WHO grade II) and anaplastic (WHO grade III) tumors. Whether children with one or other of these two variants should be stratified onto different therapeutic regimens remains contentious [5].

From the pathologist's perspective, intracranial ependymomas appear heterogeneous; there is considerable histopathological variation among tumors and within tumors, with the result that grading them in any reliable manner is difficult. Such difficulty is reflected by studies of clinically similar cohorts of children with intracranial ependymoma that report ratios of grade II to grade III tumors that range between 17:1 and 1:7, a striking discordance that likely represents both intratumoral heterogeneity, the uneven application of criteria for anaplasia by review pathologists, and idiosyncratic small patient cohorts [7]. Whether children with grade II and those with grade III ependymomas have significantly different outcomes also remains unclear; among articles with a focus on prognostic factors, those that do not show histopathological grade as an independent prognostic or predictive factor outnumber those that do [8-15,7].

Seeking to inform these difficult issues, we acquired standard histopathological preparations of ependymomas from children entered into four European clinical trials for systematic review by five neuropathologists. The review consisted of three phases: (1) grading tumors according to each pathologist's pre-study practice using the WHO classification, (2) collective evaluation of tumors from one trial cohort by all pathologists, with discussion of difficulties associated with grading, and (3) further independent review of cases following formulation of a novel grading system based on histopathological features from the WHO classification, but designed to be more prescriptive.

## Materials and methods

### Trial cohorts

**Figure 1:**
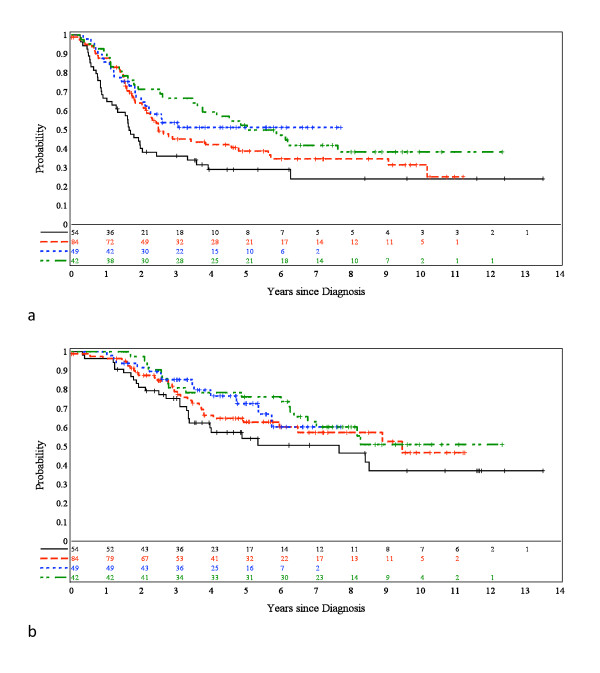
**Progression-free (PFS) and overall survival (OS) in four European trials**. (**a**) PFS and (**b**) OS by trial. Black line = SFOP; red line = CNS9204; blue line = CNS9904; and green line = AIEOP.

Ependymomas (n = 229) from children entered into four European clinical trials were requested for histopathological review, following Newcastle/North Tyneside Research Ethics Committee approval for studies on childhood brain tumors. An AIEOP trial with a postsurgical "stage-determined" protocol for non-infants provided 42 patients with a median age of 6.3 years [16]. Children on this trial were treated with (i) focal hyperfractionated radiotherapy (HFRT), if there was no evidence of post-surgical residual disease, or (ii) 4 courses of VEC followed by HFRT, if there was post-surgical residual disease. The dose of HFRT was 70.4 Gy (1.1 Gy/fraction b.i.d.), and the VEC regimen consisted of VCR 1.5 mg/m^2 ^1/w, VP16 100 mg/m^2^/day x3, and CTX 3 g/m^2^/day x1. Where feasible, second-look surgery was recommended. An SFOP trial aiming to treat young (< 5 years) children with chemotherapy alone provided 54 patients with a median age of 1.8 years [4]. Initial treatment was maximal surgical resection, which was classified as complete when post-operative neuroimaging was considered negative. Chemotherapy consisted of cycles of three courses (A: carboplatin/procarbazine, B: etoposide/cisplatin, C: vincristine/cyclophosphamide) delivered each 21 days for 7 cycles. Chemotherapy was discontinued if disease progression or unacceptable toxicity occurred. No radiotherapy was planned after completion of chemotherapy, but salvage therapy (including second-look surgery and radiotherapy) was only indicated for disease progression or a relapse. Two CCLG (UKCCSG) SIOP trials - CNS9204 and CNS9904 - provided 84 and 49 patients respectively. The CNS9204 protocol aimed to evaluate a primary chemotherapy strategy for avoiding or delaying radiotherapy in children aged less than 3 years with intracranial ependymoma [6]. Maximal surgical resection was followed by alternating blocks of myelo- and non-myelosuppressive chemotherapy every 14 days for an intended duration of 1 year. Radiotherapy was withheld unless there was recurrent disease. The overall strategy for CNS 9904 was similar to that of the AEIOP trial. After a complete surgical resection of tumor, radiotherapy (54 Gy) was delivered to the tumor site, but for those with an incomplete resection chemotherapy with VEC preceded radiotherapy (54 Gy). Across all trials, central review of all operative reports was undertaken to establish extent of surgical resection. Despite strenuous efforts, it was not possible to acquire histological preparations from all patients entered into each trial, success rates ranging from 55% (CNS9904) through 67% (AIEOP) and 74% (SFOP) to 95% (CNS9204). The clinical characteristics of children whose tumors were reviewed on this study are provided in Table 1 and are representative of those for the entire trial cohorts. Figure 1 shows progression-free survival (PFS) and overall survival (OS) for the trial cohorts used in this study.

**Table 1:** 

		SFOP	CNS9204	AIEOP	CNS9904	ALL PATIENTS
**Age at Diagnosis**	**Median**	1.8	1.9	6.3	6.8	.
	**IQR**	1.3-2.5	1.4-2.4	4.3-11.3	5.0-11.5	.
**Sex**	**Female**	28 (52%)	30 (36%)	18 (43%)	23 (47%)	99 (43%)
	**Male**	26 (48%)	54 (64%)	24 (57%)	26 (53%)	130 (57%)
**Tumor Site**	**Unknown**	.	8	.	.	8
	**Infratentorial**	45 (83%)	69 (91%)	31 (74%)	36 (74%)	181 (82%)
	**Supratentorial**	9 (17%)	7 (9%)	11 (26%)	13 (26%)	40 (18%)
**Surgical excision**	**Complete**	35 (65%)	43 (51%)	29 (69%)	19 (39%)	126 (55%)
	**Incomplete**	19 (35%)	41 (49%)	13 (31%)	30 (61%)	103 (45%)

**ALL PATIENTS**		54	84	42	49	229

### Pathology

**Figure 2:**
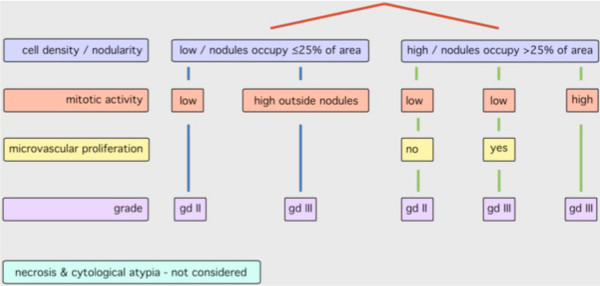
**Novel grading scheme for pediatric intracranial classic (grade II) and anaplastic (grade III) ependymomas, in which "nodules" are regions of high cell density**.

**Figure 3:**
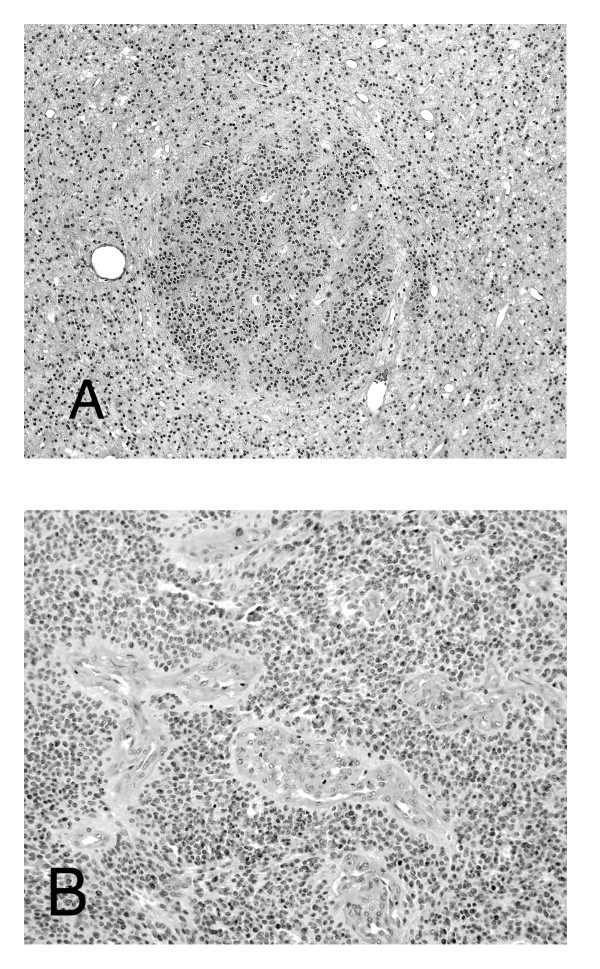
**Specific histopathological characteristics of ependymoma**. **(a) **A nodule of moderately high cell density against a low cell density background. (H&E, × 40). (**b**) The tumor vasculature here shows mural cell hyperplasia and qualifies as microvascular proliferation (H&E, ×100).

Slides of sections stained with hematoxylin and eosin (H&E) were used for pathological assessment. Ependymomas were graded according to each neuropathologist's independent interpretation of the WHO classification of CNS tumors (phase 1) or (phase 3) according to a novel grading scheme (Figure 2) that was based on a prescribed application of the WHO classification and devised by consensus following group review of all tumors from patients on CNS9204 (phase 2). The presence or absence of four histopathological features; cell density, mitotic activity, microvascular proliferation and necrosis, was recorded alongside application of the novel grading scheme. Evaluation of ependymomas according to the novel grading scheme recognized the tendency of ependymomas to show three main patterns of nuclear:cytoplasmic ratio; either low cell density, or high cell density, or nodules of high cell density within regions of low cell density (Figure 3a). Negligible (low) mitotic activity, in which situation any mitotic figure was difficult to detect across multiple high-powered fields, was distinguished from the ability to detect (high) mitotic activity by detecting at least five mitotic figures while scanning just a few high-powered fields. Microvascular proliferation was recorded as present when layers of hyperplastic mural cells, rather than just hypertrophic endothelial cells, were detected (Figure 3b).

### Statistical analysis

Graphical tools and descriptive statistics were used to describe the consensus among the five neuropathologists. Associations between clinical factors, as well as strength of consensus on histopathological variables, and progression-free survival (PFS) and overall survival (OS), were investigated using multivariable Cox proportional hazards models. P-values provided in the results section are not adjusted for multiple testing.

## Results

### Phase 1: pre-consensus grading - CNS9204, CNS9904, SFOP trials

**Figure 4:**
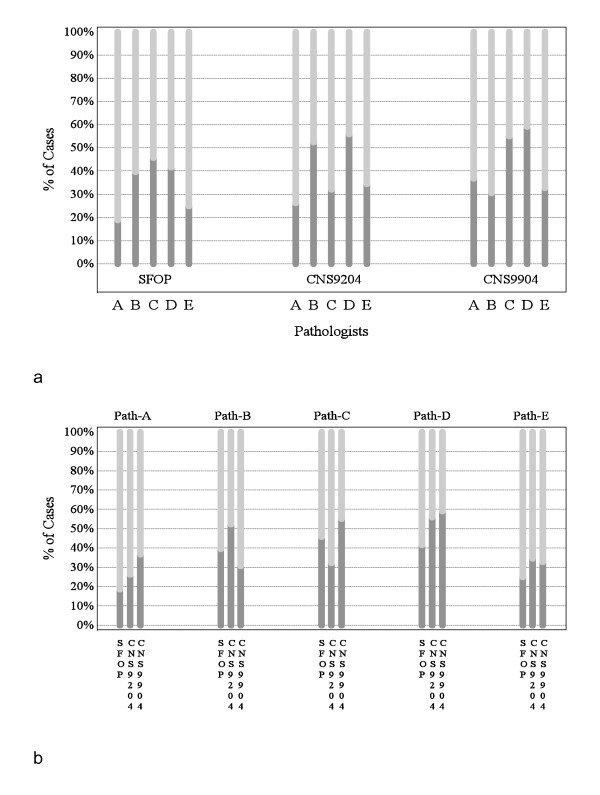
**Grading of ependymomas in three European trials by five neuropathologists (phase 1 of study)**. (**a**) Proportions of tumors classified as grade II (dark gray) or grade III (light gray) by neuropathologists A-E in phase 1 of the study, grouped by trial. (**b**) Proportions of tumors classified by each neuropathologist (Path-A, Path-B etc.) in phase 1 of the study as grade II (dark gray) or grade III (light gray).

**Figure 5:**
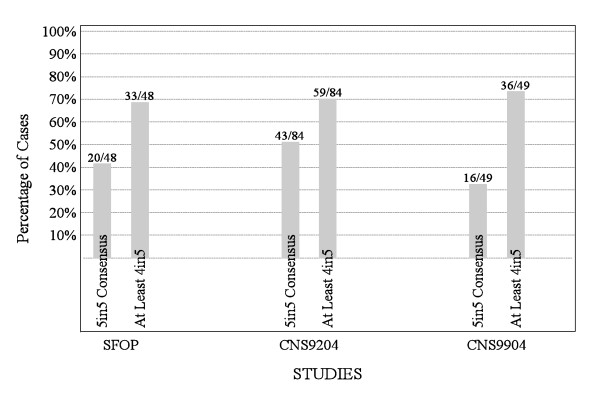
**Proportions of tumors in the three trial cohorts for which there was 4/5 or 5/5 agreement on grade among five pathologists during phase 1 of the study**.

Before any discussion of ependymoma grading, each pathologist independently evaluated tumors from children treated on the SFOP, CNS9204, and CNS9904 trials, allocating grade II or III. The proportions of ependymomas allocated grade II and grade III ranged from 19% to 59% and 41% to 81% respectively across the three trials. Figure 4 displays the ratios of grade II to grade III tumors grouped by trial (a) and pathologist (b), and Figure 5 displays agreement on grading by trial.

### Phase 2: joint review of histopathological features/grading - CNS9204 trial

**Figure 6:**
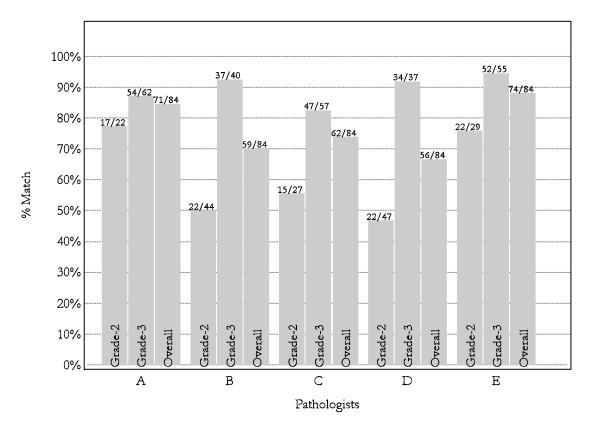
**Percentages of tumors from the CNS9204 cohort for which each neuropathologist's independent grade assignment (phase 1) matched the consensus grade after joint review by all pathologists (phase 2);** e.g. pathologist A independently classified 22/84 tumors as grade II and 62/84 tumors as grade III during phase 1, while in phase 2, joint review classified 17/22 and 54/62 as grade II and grade III tumors, respectively, producing an overall "match" on 71/84 tumors.

Joint review of all ependymomas from the CNS9204 trial cohort led to a consensus of 25 (30%) grade II and 59 (70%) grade III tumors. In achieving consensus on the grading of ependymomas from this cohort, some pathologists accepted a greater shift away from their usual practice than others (Figure 6). Following the joint review, discussion of (i) idiosyncratic cases encountered in clinical practice, and (ii) problems with evaluating types of necrosis in both grade II and grade III tumors produced further refinement of our views on grading and led to the creation of a novel grading scheme (Figure 2). This scheme emphasizes regions of high cell density, mitotic activity, and angiogenesis for grading purposes, while dismissing the influence of necrosis and cytological atypia.

### Phase 3: post-consensus grading - AIEOP trial

**Figure 7:**
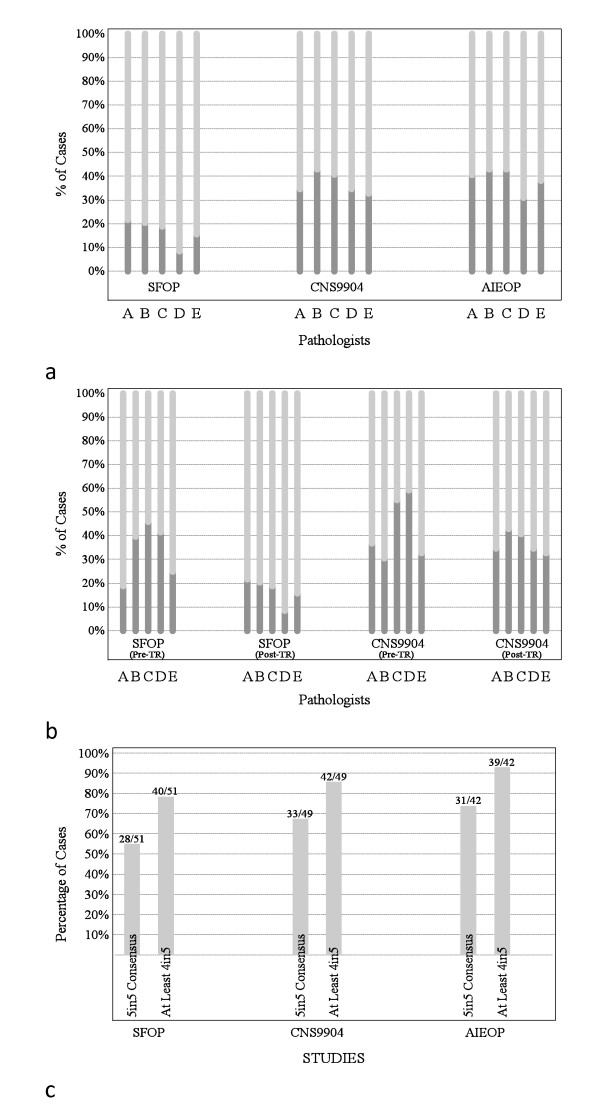
**Grading of ependymomas in three European trials by five neuropathologists (phase 3 of study)**. **(a) **Proportions of tumors classified as grade II (dark gray) or grade III (light gray) among trial cohorts. **(b) **Proportions of tumors classified as grade II (dark gray) or grade III (light gray) in two trial cohorts (SFOP & CNS9904) before (phase 1; pre-TR) or after (phase 3; post-TR) review of CNS9204 cohort in phase 2 of study (TR). **(c) **Proportions of tumors in three trial cohorts for which at least 4 of the 5 neuropathologists agreed on grade.

Ependymomas from children entered on the AIEOP trial were available for review in phase 3 of the study only. Grade II and III ependymomas represented 31%-43% and 57%-69% of tumors depending on pathologist; results that match those for CNS9904, with its similar patient population (Figure 7a).

### Phase 3: post-consensus repeat grading - CNS9904, SFOP trials

A second independent evaluation of ependymomas from CNS9904 and SFOP based on the new classification allowed us to assess concordance on grading among pathologists before and after consensus was reached on the creation of a novel grading scheme. In both trial cohorts, there was a substantial improvement in agreement on grades (Figures 5, 7b, and 7c); cases for which there was perfect agreement increased from 33% to 67% in CNS9904 (p = 0.0001) and from 42% to 55% in SFOP (p = 0.006). Cases for which there was perfect agreement or only one dissenter increased from 73% to 86% in CNS9904 (p = 0.065) and 69% to 78% in SFOP (p = 0.013).

### Post-consensus concordance on histopathological variables

**Figure 8:**
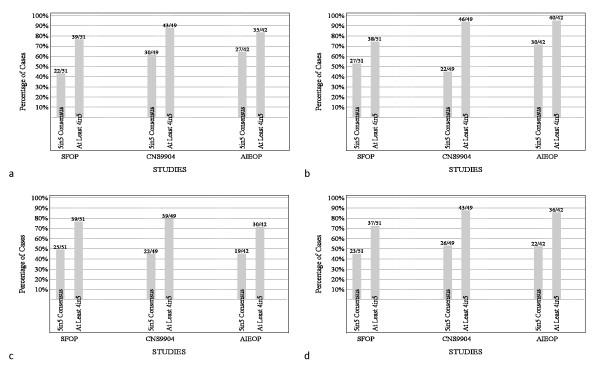
**Strength of consensus among neuropathologists on histological variables**. Consensus on **(a) **cell density, **(b) **mitotic activity, **(c) **microvascular proliferation, and **(d) **necrosis was closely matched across studies during phase 3, but was lower for angiogenesis than for other variables.

Cell density, mitotic activity, angiogenesis, and necrosis were histopathological variables assessed as part of the phase 3 review of ependymomas from children on the CNS9904, SFOP, and AIEOP trials. Strength of consensus was closely matched across studies, but was lower for angiogenesis than other variables (Figures 8a-d).

### Association between outcome and clinical variables

For each trial, outcome analyses were undertaken using clinical and pathological data. The following clinical variables were considered: age at diagnosis, gender, tumor site, and surgery extent. PFS and OS were significantly associated with extent of surgical resection in the AIEOP (PFS: p = 0.003; OS: p = 0.014) and SFOP (PFS: p = 0.003; OS: p = 0.016) cohorts. In the SFOP cohort, OS was also significantly associated with tumor site; infratentorial tumors were associated with a worse outcome (p = 0.025). In CNS9204, age at diagnosis was associated with OS, young age being correlated with a worse outcome (p = 0.012). No other clinical variable was associated with outcome in CNS9204, and none at all in CNS9904.

### Association between outcome and concordance on grading

Data from phase 1 of the study, when ependymomas from trial cohorts CNS9204, SFOP, and CNS9904 were graded independently by each of the pathologists according to their usual diagnostic practice, revealed no association between strength of consensus on grade and outcome.

In phase 2 of the study using trial cohort CNS9204, when tumor grade and the status of 4 histopathological features were agreed by all 5 pathologists around a multi-headed microscope, no pathological variable was shown to be prognostic indicator.

**Figure 9:**
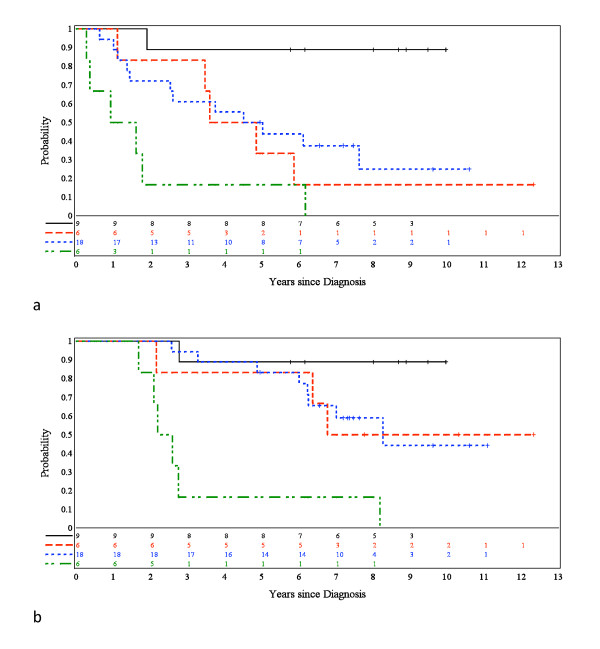
**Progression-free (PFS) and overall survival (OS) in AIEOP trial cohort patients split according to histological grade and extent of surgical resection, showing significantly different survival curves (PFS p = 0.0004; OS p < 0.0001)**. **(a) **PFS and **(b) **OS. CR = complete resection; STR = subtotal resection; black line = CR-Gd. II; red line = STR-Gd. II; blue line = CR-Gd. III; green line = STR-Gd. III

When ependymomas from CNS9904, SFOP and AIEOP were evaluated independently by each pathologist in phase 3 of the study, there was no association between strength of consensus among pathologists on grade and PFS for children treated on CNS9904 and SFOP. Similarly, there was no association between PFS for children treated on CNS9904 and SFOP and the strength of consensus among pathologists on the status of any individual histopathological feature. In contrast, strength of consensus among pathologists on grade and on every histopathological variable was significantly correlated with PFS in the AIEOP trial cohort. Combinations of consensus grade and extent of surgical resection produced significantly different survival curves (PFS and OS) when examined in the AIEOP cohort only (Figure 9).

The above results were unchanged, if instead of degree of consensus, the calls of individual pathologists on grade and histopathological variables were analyzed against outcome. Overall, no one pathologist's approach to the assessment of ependymomas prevailed as a correlate of biological behavior.

## Discussion

No previous international study has systematically addressed the histopathological evaluation of ependymomas in the manner reported here, providing data on the review of 229 intracranial ependymomas from children entered into 4 European trials. The review was conducted by 5 neuropathologists, all with specialist experience in the field of pediatric neuro-oncology. The final phase of the study (phase 3) employed a novel histopathological grading scheme. This represents a prescribed application of the WHO classification and was derived by consensus from both the pathologists' experience of ependymomas and a joint review of tumors from one of the four trials (CNS9204). Key aims of the study were: (i) to assess whether discussion surrounding the conception of the new grading scheme and its principles could be used to improve concordance on grading among pathologists, and (ii) whether any pathological variable, either grade itself or the status of one of four histopathological features, was associated with outcome in the setting of formal ependymoma clinical trials with different therapeutic approaches.

Our study was prompted by lack of consensus on how to grade childhood intracranial ependymomas; a huge discrepancy exists between the ratio of grade II:III tumors across the literature, and there is considerable scepticism as to whether grading intracranial ependymomas has clinical utility [7]. The 2007 WHO classification distinguishes the anaplastic (grade III) from classic (grade II) ependymoma on the basis of "high mitotic activity, often accompanied by microvascular proliferation and pseudopalisading necrosis" [1]. This reflects a general principle of the pathological assessment of gliomas - that the identification of 'anaplastic' features, such as increased cell density, mitotic activity, microvascular proliferation, and necrosis, can be used to derive a clinically useful grade. In diffuse astrocytic tumors, these features are progressively acquired with increasing grade (fibrillary astrocytoma, grade II - anaplastic astrocytoma, grade III - glioblastoma, grade IV) and are recognized prognostic indicators [17,1]. Reinforcing the biological relevance of histopathological grading, astrocytoma progression is associated with the acquisition of specific genetic abnormalities [1]. In contrast, anaplastic ependymomas tend to present *de novo*; it is uncommon for recurrent ependymomas progressively to acquire an anaplastic phenotype, and any genetic basis for this phenomenon has not yet been convincingly demonstrated [18]. In addition, the presence of anaplastic features across an ependymoma is notoriously variable in magnitude and extent, potentially making evaluation of these features difficult and subsequent grading subjective. For example, a pathologist may be faced with a small focus of microvascular proliferation or pseudopalisading necrosis in a tumor devoid of mitotic activity and with a low cell density. Should this discovery prompt a diagnosis of anaplastic ependymoma (grade III), or should the dominant grade II phenotype prevail?

In phase 1 of this study and before discussing the grading of ependymomas, the five study neuropathologists showed only fair concordance for grade among the group, while showing individual consistency across trials. If ependymomas are particularly difficult tumors to grade, it is surprising that the levels of inter-observer concordance recorded in this study are not far removed from those reported for other gliomas. Assessing astrocytomas and oligodendrogliomas, Coons and colleagues reported an initial 4-reviewer concordance on grade of 52%, which compares with 51% for 5/5 consensus on grading in CNS9204 in this study [19]. Mirroring our experience, concordance improved over successive reviews, as their pathologists discussed possible explanations for discrepancies and developed criteria to aid grading. Grade was assigned according to the status of the same histopathological variables used in the present study, among which microvascular proliferation proved hardest to evaluate in both studies, with lower levels of agreement on its status than for other histopathological features.

After discussing the problems of grading ependymomas and devising a novel grading scheme (phase 2), our study pathologists assessed ependymomas from two trial cohorts for a second time at an interval of just over one year (phase 3). Concordance on grading was notably improved at this time, though it was apparent that some pathologists altered their practice to accommodate the new scheme more than others. This outcome does not necessarily suggest that the new grading scheme presented here is better than the WHO classification, to which the neuropathologists were working in phase 1 of the study, just that agreement to work to a scheme in a prescribed manner results in increased concordance. However, one corollary of the improvement could be that it is easier to grade ependymomas consistently using a more detailed and prescribed scheme than the current WHO classification.

Various clinical variables have been associated with outcome in trial cohorts of children with intracranial ependymoma. These include age at presentation, tumor location, and extent of surgical resection [9,4,15,20-24]. In addition, there is undoubted evidence to indicate the benefits of radiotherapy [5,3,14]. A trend towards shorter PFS and OS in the infant cohorts (SFOP/CNS9204) was observed, but in the present study a significant positive association between age and OS (but not PFS) was observed only among children from the CNS9204 cohort. Infratentorial tumor location was significantly associated with poorer OS (but not PFS) only among children from the SFOP cohort. Extent of surgical resection, which has been a proven prognostic indicator in most studies of pediatric ependymoma [22,23], was associated with outcome in only two (SFOP/AIEOP) out of four of the present trial cohorts, and it may be relevant that the proportion of completely excised tumors in these trial cohorts (SFOP = 65%; AIEOP = 69%) is greater than in either the CNS9204 (51%) or CNS9904 (39%) trial.

The study design enabled us to examine potential associations between outcome and multiple histopathological features. With assessments from five pathologists, it was also possible to analyze the relationship between outcome and strength of consensus among pathologists on grade or the status of individual histopathological features. Adjusted for extent of surgical resection, strength of consensus on grade and on each of the histopathological features was significantly associated with PFS in the AIEOP cohort, but in none of the other cohorts. When individual pathologists' calls on these variables were reviewed; grade III, high cell density, high mitotic activity, presence of microvascular proliferation, and presence of necrosis were all significant adverse prognostic indicators for the AIEOP cohort, but not for SFOP or CNS9904. In the setting of satisfactory concordance on histopathological interpretation, our results suggest that grading ependymomas might have clinical utility either in older children (versus infants) or in children that have received radiotherapy immediately post-surgery (usually non-infants). The latter conclusion is supported by an association between ependymoma grade and outcome in an extensive study of children of all ages, including infants, that were treated with radiotherapy soon after surgery [25]. However, a major caveat from our study involves the lack of a similar association in children from the CNS9904 cohort. These discrepant findings might be related to different proportions of completely versus incompletely resected tumors in the AIEOP (69% vs. 31%) and CNS9904 (39% vs. 61%) cohorts. There are few fundamental differences between studied cohorts to explain our data; there was a significant association between extent of surgical resection and outcome in the AIEOP and SFOP but not the CNS9204 and CNS9904 series, and irradiation as a first line treatment was incorporated into the AIEOP, but not SFOP, trial.

Our data also suggest that a more robust assessment of ependymomas for therapeutic purposes may await the application of a more sophisticated combination of histological and molecular approaches, but while markers of tumor cell proliferation, e.g. Ki-67 immunolabeling [9,26-28], and a few molecular abnormalities, e.g. copy number gain across chromosome 1q and ERBB2/4 receptor expression [29-32], have been proposed as prognostic indicators, insufficiently unambiguous data are available from large patient cohorts for the confident creation of such a scheme. It is also possible that continuing difficulties with finding prognostic markers for this tumor reflect a misplaced view of ependymomas as homogeneous across the neuraxis, a principle implicit in the WHO classification, which does not differentiate between supratentorial and posterior fossa tumors. However, both data showing that ependymomas at these two sites are characterized by distinct gene expression profiles and identification of a 'vascular' variant in a mainly supratentorial location make the case that ependymomas from these sites should probably be appraised separately [33,34].

## Conclusions

We have provided a substantial amount of data on the histopathological evaluation of pediatric intracranial ependymoma and its clinical associations. Being more prescribed than the latest WHO classification in its approach to grading ependymomas, the novel scheme we describe may offer advantages with respect to reproducibility and its ability to link the pathology of ependymomas to outcome, and we would welcome more studies of its effectiveness in other ependymoma trial cohorts.

## Competing interests

The authors declare that they have no competing interests.

## Authors' contributions

DE conceived the study and contributed to its design, pathology review, data collection and data analysis, and drafted the manuscript. MK and JB contributed to data analysis. DF-B, FG, CG, and TP contributed to study design and undertook pathology review. DF, MM, JG, and RG contributed to study design and data collection. All authors read and approved the final manuscript.
